# Favoring the cognitive-motor process in the closed-loop of BCI mediated post stroke motor function recovery: challenges and approaches

**DOI:** 10.3389/fnbot.2023.1271967

**Published:** 2023-10-10

**Authors:** Jing Mang, Zhuo Xu, YingBin Qi, Ting Zhang

**Affiliations:** ^1^Department of Neurology, China-Japan Union Hospital of Jilin University, Changchun, China; ^2^Department of Rehabilitation, China-Japan Union Hospital of Jilin University, Changchun, China; ^3^Department of Neurology, Jilin Province People's Hospital, Changchun, China; ^4^Rehabilitation Therapeutics, School of Nursing, Jilin University, Changchun, China

**Keywords:** stroke, rehabilitation, motor control, cognitive function, brain-computer interface

## Abstract

The brain-computer interface (BCI)-mediated rehabilitation is emerging as a solution to restore motor skills in paretic patients after stroke. In the human brain, cortical motor neurons not only fire when actions are carried out but are also activated in a wired manner through many cognitive processes related to movement such as imagining, perceiving, and observing the actions. Moreover, the recruitment of motor cortexes can usually be regulated by environmental conditions, forming a closed-loop through neurofeedback. However, this cognitive-motor control loop is often interrupted by the impairment of stroke. The requirement to bridge the stroke-induced gap in the motor control loop is promoting the evolution of the BCI-based motor rehabilitation system and, notably posing many challenges regarding the disease-specific process of post stroke motor function recovery. This review aimed to map the current literature surrounding the new progress in BCI-mediated post stroke motor function recovery involved with cognitive aspect, particularly in how it refired and rewired the neural circuit of motor control through motor learning along with the BCI-centric closed-loop.

## 1. Introduction

Recovery of motor function is of significant importance for physical independence and social integration of stroke patients. One of the ambitions of post stroke motor function restoring (PMFR) is to recouple the brain and external muscles while supporting the patients' personhood by regaining functional activities of daily living (e.g., walking, gait) rather than replacing incomplete limbs with lifelong prostheses (Kübler, 2020). It is widely accepted that restore of motor function in patients with stroke is based on the exploitation of neuroplasticity, which promotes the reconstruction of the motor control system through motor learning (Teasell et al., 2014).

“The cortical motor system is not an unthinking, passive circuit controlled by more intelligent parts of the brain (Kandel et al., 2000).” Unlike what we commonly think of as the simple generation of a series of muscle activities, voluntary motor control is understood in a broader sense as a process that is more sensory, perceptual, and cognitive in nature (Chivukula et al., 2019; Sensinger and Dosen, 2020). Therefore, the cognitive processes of motor control and motor learning in stroke survivors underpin substantial gains in the PMFR. In recent decades, the neuroscientific theory of the inseparable cognitive processes involved in motor control and motor learning has further extended the boundaries of post stroke rehabilitation strategies, such as constraint-induced movement therapy, mirror therapy, motor imagery, enriching environment, etc. (Ward, 2017; Maier et al., 2019). However, there are still many applicable gaps in translating neuroscientific principles into protocols of PMFR. The reason may be that traditional rehabilitation treatment methods may attach importance to only one or several nodes in the motor control loop and lack the function of forming a unified and complete motor pathway. Therefore, the ideal strategy might be to build up a continuous loop to “fire together and wire together” consistent with the incomplete or impaired movement control circuit after stroke, which can reinforce the motor control through motor learning.

**Figure 1:**
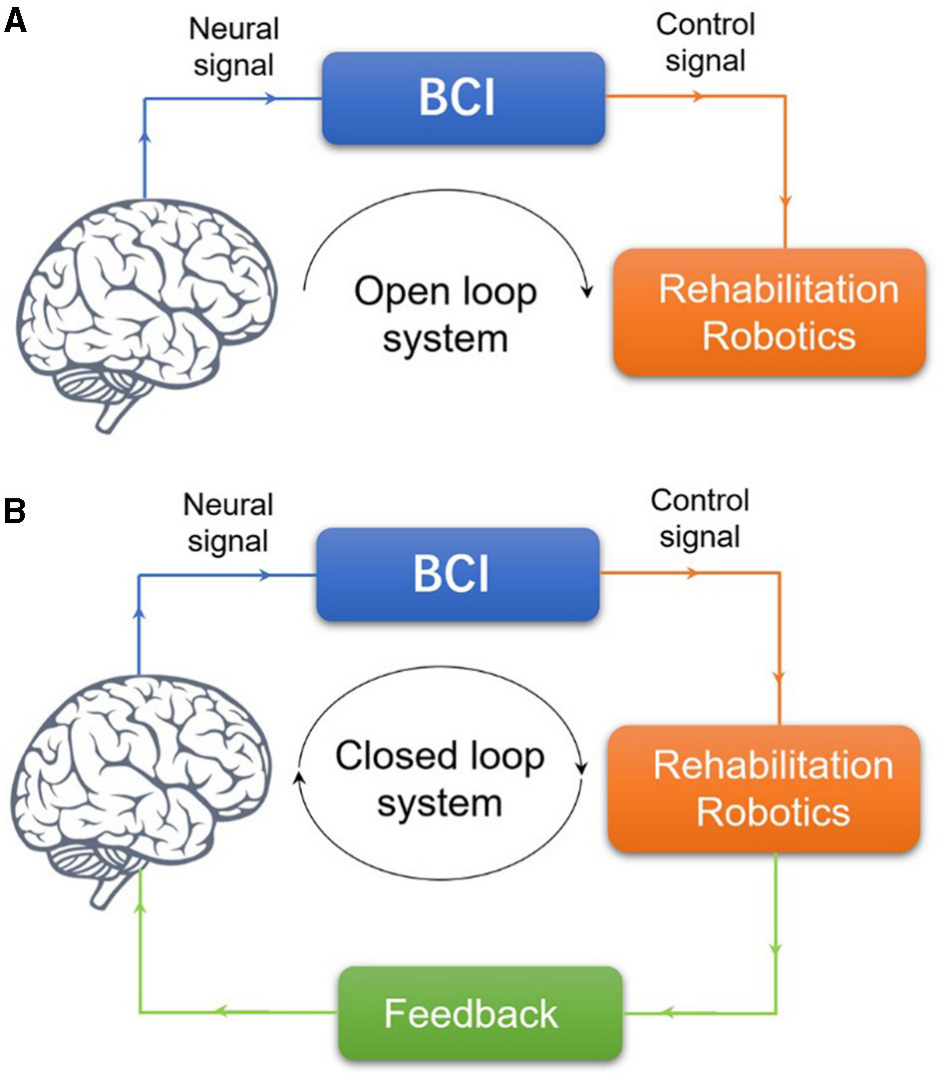
BCI system for PMFR. **(A)** Illustration of the concepts of open loop BCI system. **(B)** Illustration of the concepts of closed-loop BCI system.

With the boom in robotic technologies, robot-assisted therapy is now seen as promising to compensate for the innate disadvantages of traditional physical and occupational therapy, turning more, and more theoretical insights into real applications. In the field of human-robot cognitive interface, concerns have been expressed about what a robot, based on neuroscientific principles, can do in motor rehabilitation after stroke to synergy the conventional therapy rather than merely replacement. The most convenient and widely used noninvasive method to connect the brain to an assistive device is the EEG-based Brain-Computer Interface (BCI; Birbaumer et al., 2008; Sitaram et al., 2017). Regarding clinical aspects, this review has mainly focused on EEG-based BCI systems. Over the past decades, the BCI has gradually taken robots beyond the tools passively used by therapists. For example, a BCI can supplement stroke survivors' impaired muscle control by decoding their motor intentions into signals to manipulate external devices such as neuroprostheses or exoskeletons, which the BCI system operates in an “open loop.” As a counterpart or compensation to the “open loop,” in the “closed-loop” manner, the end-user controls the rehabilitation robotics and receives sensory feedback provided by the BCI system to promote stimulatory neuroplasticity-based reorganization of the motor-related brain regions (Wang et al., 2010; see Figure 1).

Interestingly, the idea of a closed-loop BCI system is in line with the way that the natural human motor control circuit works. Within this loop, state-of-the-art approaches have been developed at various nodes with profound benefits and challenges. Namely, from simple brain signal extraction devices to their combination with functional electric stimulation devices, from somatic sensory feedback alone to multimodal environmental conditioning, from simple assistive robotics to adapted devices with feedback sensors and timely external stimulations, and so on. All these innovations and add-on interventions are enhancing the theoretical and methodological development of the closed-loop non-invasive BCI, which could promote neuroplasticity through the embodied cognitive process of the human side. A comprehensive understanding of these issues could advance the engineering and design of robot-aided PMFR approaches. This review provides a focused overview of the progresses that facilitate the cognitive-motor circuit within the closed-loop of EEG-based BCI systems designed to promote PMFR.

## 2. Rethinking the cognitive aspects on the human side in the context of BCI-mediated PMFR

**Figure 2:**
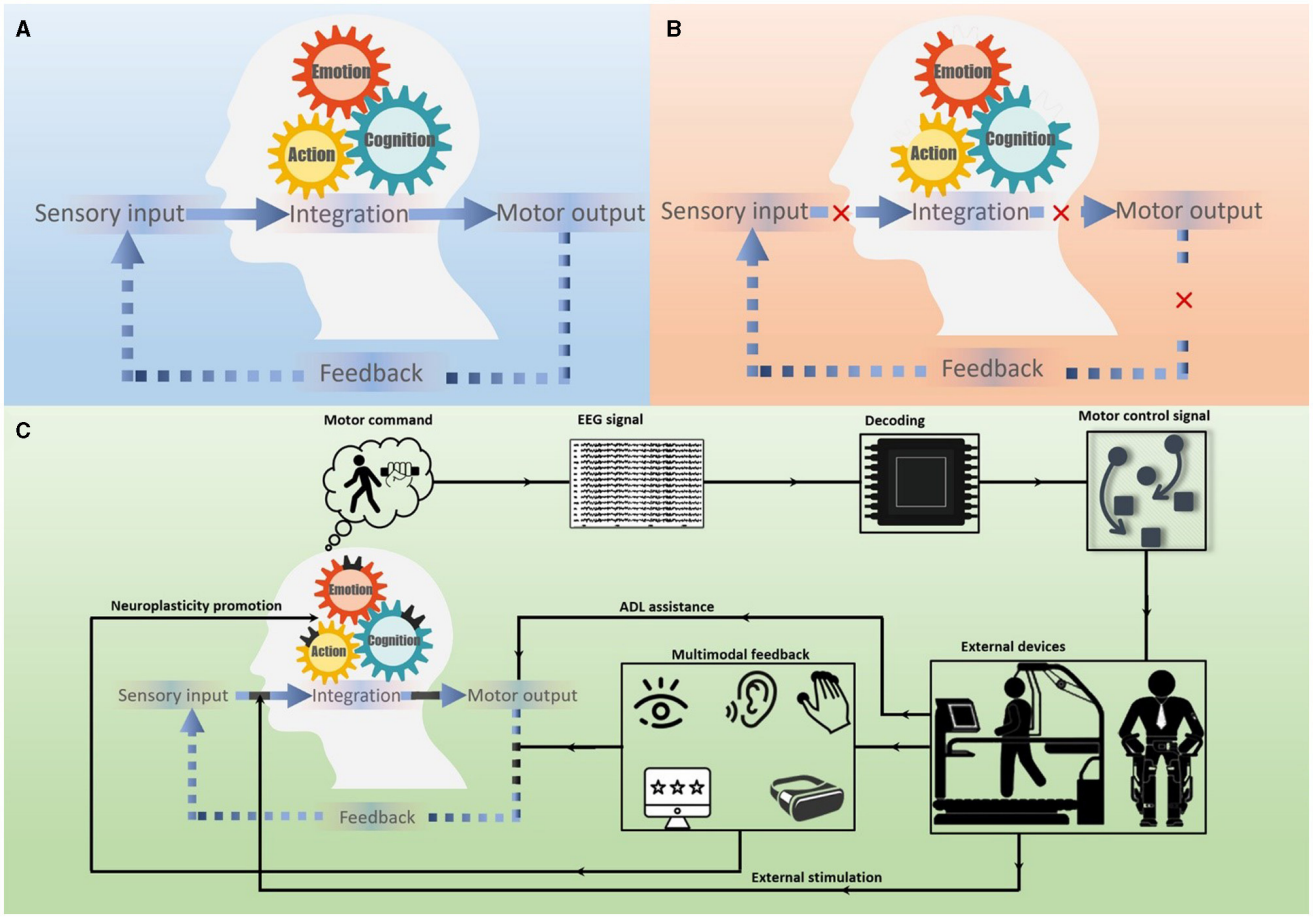
Schematic of the closed-loop BCI system for PMFR. **(A)** The normal cognitive-motor control loop. **(B)** The cognitive-motor control loop interrupted by stroke. **(C)** The closed-loop BCI system for PMFR. The BCI-mediated PMFR was based on the MI-EEG system, provided multimodal feedback to the subject, and formed a closed-loop that promoted motor learning through neuroplasticity as well as assisted patients with activities of daily living (ADL).

The human motor control system does not simply consist of unthinking or passive circuits. The motor and cognitive functions interwove in a seamless fashion in the motor control loop. In addition to the primary motor cortex, many areas [e.g., supplementary motor areas (SMA), cingulate motor areas (CMA), premotor areas (PMA)] in the frontal lobe and parietal lobe are wired together to participate in motor control during self-paced movements (Rizzolatti and Luppino, 2001; see Figure 2). In a certain number of stroke patients, the motor function deficiency is due to the impairment of cognition or mental processing, which is anchored in action (Platz et al., 2000). Furthermore, even in the ipsilateral motor, both the two cortical hemispheres are interconnected through the corpus callosum. These neural circuits are responsible for controlling voluntary behavior rather than simply generating a particular pattern of muscle activity. The context-depended paradigm of the activation and deactivation of the motor control system can be presented as event-related desynchronization and synchronization (ERD/ERS) of sensorimotor rhythms (SMR), which can be detected and recorded by electrodes placed on the scalp (EEG) or directly on the exposed surface of the brain (ECoG; Georgopoulos, 2000; Rizzolatti and Luppino, 2001). It should be noted that the effects on the cognitive process of the cerebral cortex may be individualized, i.e., a patient with locked-in syndrome could have intact cognition but complete loss of voluntary movement (Plum and Posner, 1982). Nonetheless, the conceptual issues regarding the cognitive features of motor physiology after stroke should be considered for the rationale and heuristic value for the scope of investigation of BCI-mediated PMFR. In addition, other cognitive features of the human side in the process of PMFR should be considered when speaking of a closed BCI loop.

### 2.1. Somatosensory information from the body

Sensory feedback plays an important role to maintain motor cortical activity and circuitry (Tabot et al., 2015; Carteron et al., 2016). Motor control encompasses mainly a process of sensor input and motor output. There is not only a control mechanism for the execution of the movement, but also for whether, how, and when to act. The somatic sensory input from body receptors can act as feed forward control of intended movement and feedback control of ongoing movements. In stroke patients, a loss or weak of proprioception and tactile sensation is the most likely to occur due to impairment on the somatosensory pathway. In addition, this somatosensory loss can also induce the disuse of paretic limbs after stroke (Dannenbaum and Dykes, 1988). Studies in humans and non-human primates have shown that the stroke-impaired limbs might be continued to disuse even when their capacity are recovering, which is referred to as the “learned non-use phenomenon (Han et al., 2013; Taub et al., 2014).” The compensatory use of the non-paretic limb may limit the subsequent gains in motor function in the paretic limbs (Oujamaa et al., 2009). Interestingly, reducing somatosensory input from the intact hand may serve as another solution to the learned nonuse phenomenon of the paretic limbs, just as in constraint-induced movement therapy (CIMT; Sens et al., 2013). Moreover, in the BCI-mediated system, the somatosensory-induced effects can be augmented by the sensory components of the external robotic device, which offered a greater advantage of incorporating proprioception and tactile feedback to the users.

### 2.2. Sensorimotor interface in the human brain

The sensorimotor interface, which relays sensory input to higher motor control areas, plays an important role in the integrity of the motor control circuit. The posterior parietal cortex (PPC) has been recognized as an association cortex in the sensorimotor pathway. The PPC is involved in motor intention, movement planning, spatial reasoning, and integration of multisensory feedback that is transmitted to the frontal lobe for movement control. Damage to the PPC from stroke can impair the patient's ability to plan movements and perceive spatial relationships that affect sensorimotor circuitry (Buneo and Andersen, 2006). The damaged sensorimotor interface of stroke survivors can be both spatial and temporal and has important implications for the development of BCI-driven PMFR (Mihara et al., 2012). In terms of a closed-loop, the sensory side of the motor control system can be greatly compensated by multisensory feedback provided by BCI-controlled external devices (Bolognini et al., 2016).

### 2.3. Motor imagery/observation ability

Based on the theory of mirror neuron system, mirror therapy, action observation, and motor imagery therapy have been widely explored in conventional motor rehabilitation practice (Iacoboni and Mazziotta, 2007). Motor imagery (MI) can be defined as mental rehearsal of a certain movement without physical performance. As a self-paced mental practice to improve motor performance, MI has been gained prominence as a trigger of motor commands for BCI-mediated PMFR. Studies have shown that ERD/ERS can be intercepted in both the process of physical action and MI in healthy subjects (Pfurtscheller et al., 2006). This neural basis provides an opportunity for further research, i.e., even if the stroke-affected limb was too weak to move to generate adequate motor signals, BCI can alternatively use brain signals generated from MI. Many studies have demonstrated the efficacy of MI-based BCI technology in robotic rehabilitation. However, not all stroke survivors can reliably perform MI due to impaired cortex function. Interestingly, however, the impaired motor imagery ability seems to prevent patients only slightly from successfully operating the MI-BCI system (Ang et al., 2011; Braun et al., 2017). The firing of the mirror neuron system during action observation can also be reduced after stroke, EEG studies found that Mu suppression (attenuation in alpha band power strength) recorded over the sensorimotor cortex during action observation was reduced in the stroke-affected hemisphere (Frenkel-Toledo et al., 2014). The characteristics of these results suggested that it is imperative to assess the motor imagery/observation ability prior to related BCI procedures.

### 2.4. Mental status

Approximately one-third of post-stroke patients suffered mood disorders such as depression, anxiety, stress, etc. Although the clinical manifestations may be a mixture of these disorders, the post stroke depression (PSD) has been the most extensively studied and reported in the literature. The emotional changes in stroke patients can be a result of the brain physical damage to the mood regulation systems (Grajny et al., 2016), but more common be a result of difficult or traumatic life experiences (e.g., post-stroke disability, unsatisfactory treatment outcomes, too difficult rehabilitation goals, etc.). The hallmark of PSD that threatens PMFR is a lack of motor motivation, resulting in less patient participation in rehabilitation activities (Nannetti et al., 2005). Even the true neurobiological mechanism behind post-stroke emotional disturbance cannot be elucidated in the available scientific evidence, but it does indeed cause the motor control circuitry to operate in a retarded state. Nevertheless, solving this problem again opens a window for the utility of BCIs (Dannenbaum and Dykes, 1988).

### 2.5. Task-specific and environmental condition exercise

In terms of PMFR, task ability is more significant than movement performance without goal guidance. The fulfillment of the rehabilitation goal should not rely only on the ability of the remaining motor neurons or motor cortexes to fire, but rather on the connection or the ability of the connectivity of the elements to perform the motor output as task accomplishment (Young et al., 2014). From a neuroscience perspective, task-specific training refers to a voluntary behavioral process that integrates information from the environment and translates intention into a series of actions, which appears to be perceptual and cognitive rather than purely motor in nature. There is a growing body of evidence supporting the efficacy of intensive task-specific (Jeffers et al., 2020). However, the regained ability to perform a task in the vicinity of a clinic or laboratory may not improve performance in a patient's daily life at home. One explanation is that sensory feedback from environmental factors plays an important role in inducing the optimal response of stroke survivors. Thus, separating motor skills from an individual's integrated function or separating the task from the corresponding environment in task-specific training may be simplistic and incorrect (Sigrist et al., 2015). The neural rationale of task-specific training for functional recovery after stroke involves the neuroplasticity that occurs in many brain parts and circuits that perform the function of selecting, planning, and even inhibiting motor actions. According to imaging studies, these areas include the parietal lobe, precentral motor cortex, visual cortex, and associated subcortical pathways, etc. Neuroimaging findings in animals suggest that injuries in unique regions may be related to impairment in a particular task after stroke (Jeffers et al., 2020). Although there is limited evidence of such anchored mapping in humans, this leaves room for further studies of region-specific BCIs in conjunction with task-specific training for stroke survivors.

## 3. Discriminating the brain signal for BCI mediated PMFR

As a starting point of the closed-loop in BCI-mediated PMFR, the acquisition methods of brain activity signals can currently be divided into noninvasive and invasive. Non-invasive methods include electrocorticography (EEG), functional magnetic resonance imaging (fMRI), magneto-encephalo-graphy (MEG), and near-infrared spectroscopy (NIRS), while invasive methods include electrocorticogram (EcoG) and intracortical decoding with penetrating electrodes (Tam et al., 2019). Surface EEG is commonly used in BCI because of its high temporal resolution, cost-effectiveness, transferability, and non-invasiveness (Teo and Chew, 2014). Among BCI studies using EEG methods, sensorimotor rhythm (SMR) is the most commonly used signal to control external devices, which is discussed in this paper with a focus on protocol modification and technology advancement (Hwang et al., 2013).

### 3.1. Fundamental principles of SMR-based EEG

EEG is a method that measures electrical signals from the brain at the surface of the scalp. Traditionally, EEG signals are divided into several frequency bands, including δ (0–4 Hz), θ (4–7.5 Hz), α (8–13 Hz), β (13–30 Hz), γ (30–100 Hz). Of these bands, the most important for movement decoding is the oscillation in the alpha band in sensorimotor cortex, also known as μ-rhythm (Chatrian et al., 1959; Schomer and Lopes da Silva, 2017). It has been shown that the signal power in the alpha band decreases when subjects engage in motor execution or imagery, and similar changes are observed in the beta band (Yuan and He, 2014). SMR is the modulation of the signal band power in the sensorimotor region. The reduction in band power coincides with the event is called event-related desynchronization (ERD). In contrast to ERD, event-related synchronization (ERS) is the increase of band power that coincides with an event (Tam et al., 2019). ERD changes usually begin before movement, are concentrated in the contralateral sensorimotor region, and then spread to the ipsilateral side, becoming bilaterally symmetric before movement onset, and remaining bilaterally symmetric during movement. After movement ceases, ERS changes may manifest as increased beta band power in the contralateral sensorimotor areas, also referred to as “beta rebound” (Graimann et al., 2002).

### 3.2. Neural aspects related to resolution of EEG

Due to the potential complexity and non-stationarity of EEG, the accuracy of BCI control still needs to be improved. One of the most critical signal processing steps in SMR-based motor decoding is the estimation of signal power in the α and β bands. There are various techniques to achieve this. One of the simplest and most efficient methods is band-pass filtering (Tam et al., 2011). The adaptive auto-regressive model (AAR) is another widely used detection technique that can help to choose the most appropriate frequency band to perform the filter (McFarland and Wolpaw, 2008). In this context, recent studies have identified many aspects that have the potential to improve the accuracy of EEG.

#### 3.2.1. Neuronal populations

**Figure 3:**
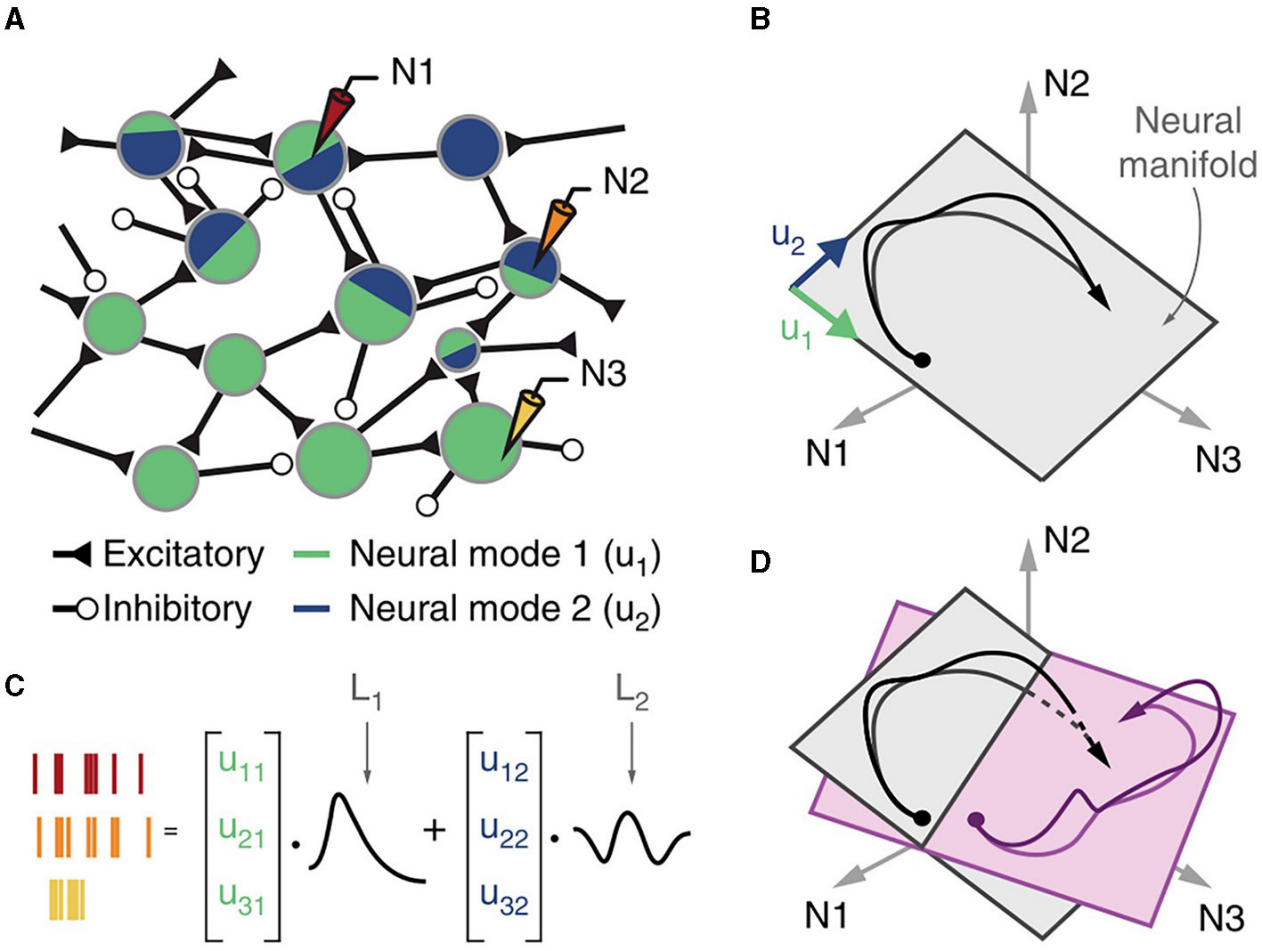
Cortical population activity within a preserved neural manifold. **(A)** The connectivity of the cortical network leads to neuronal modes. **(B)** The neural space for the three neurons (N1, N2, and N3). The time-dependent population activity is represented by a trajectory (in black, the arrow indicates time direction) mostly confined to a two-dimensional neural manifold (gray plane) spanned by two neural modes (green u 1 and blue u 2 basis vectors). **(C)** The time-dependent activity of each recorded neuron is a weighted combination of the latent activities L 1 and L 2, each the time-dependent activation of the corresponding neural mode. **(D)** Neural manifolds for different tasks (shown in gray and light purple) may have similar orientation, and the latent activities for the two tasks (shown in black and purple) may be similar.

A better understanding of the firing pattern during motor actions is critical for developing more effective signal extraction and decoding strategies. Pervious brain function studies have focused on the correlation between single-neuron activity and associated behavior, but further studies have shown that many corticomotoneuronal cells do not represent specific movement covariates at the level of single neurons (Fetz et al., 1989). Consequently, extracting information from neuronal populations activated during a particular movement becomes an urgent problem to be solved in the field of EEG-based BCI (Pfurtscheller et al., 2000). A major advance of neuroscience in this area lies in the proposals of “neural modes” and “neural manifold.” The definition of manifold comes from computational neuroscience, which states that the underlying network connectivity constrains the possible patterns of activity of neuronal populations. These patterns are restricted to a low-dimensional manifold spanned by a few independent variables called “neural modes” (Gallego et al., 2018). The neural manifold means neuronal population activity tends to be in low-dimensional space (Figure 3). Using neural population activity to reflect the user's motor intention, manifold-based EEG stabilizers can offer significant advantages over existing methods for keeping the BCI systems stable under parameter fluctuations (Gallego et al., 2018; Degenhart et al., 2020).

#### 3.2.2. Cognitive disconnection

In MI-based BCIs, a relatively low spatial resolution of the EEG may not match the complexity of MI tasks, leading to cognitive disconnection during BCI operation. To address this problem, source-based EEG approaches have been explored to divide complex motor tasks into different manipulations, e.g., subdivision of hand movement into flexion, extension, supination, and pronation (Edelman et al., 2015). More recently, offline datasets that decode different phases of the motor task with different joints of the same limb showed promise for facilitating the operation of MI-based BCI and reducing the cognitive load on users (Ma et al., 2020).

#### 3.2.3. Hand dominance

In healthy subjects, lateralization of SMR during motor imagery was observed to be associated with handedness. Left-handers showed lower accuracy in BCI performance and poorer SMR reduction in the alpha band (8–13 Hz) during mental simulation of left-handed movements (Zapała et al., 2020).

### 3.3. Progress in EEG hardware technology

Currently, EEG is widely used in BCI, but the stability and accuracy of EEG signals still needs further improvement due to the instability of brain activity and susceptibility to environmental artifacts. It should be noted that motor control processes are not rigidly compartmentalized into distinctive neural structures or neural populations. A major challenge in extracting motor brain signals is mapping the topographic representation of different body parts, which increases the difficulty of the practical application of EEG in BCI. On the other hand, the bottlenecks of EEG technology itself need to be further solved, e.g., the volume conduction effect, the stability of electrodes, the portability of devices, etc.

#### 3.3.1. High-density EEG

As a promising technique for brain signal extraction, high-density electroencephalography (hdEEG) has been used for signal acquisition during BCI operation, benefiting from its high spatial and spectral resolution (Liu et al., 2017). The hdEEG often has 256 channels or even more, compared to conventional low-density EEG (32–64 channels). It can help to study the neural signatures of hand, foot, and even lip movements in more detail (Zhao et al., 2019).

#### 3.3.2. Wireless EEG

Recently, wireless solutions are transforming traditional stationary EEG systems into portable wireless systems with high signal quality (Mihajlović et al., 2014). A wireless EEG system is established commonly based on the Bluetooth or WIFI technology (Zhang et al., 2014). The wireless EEG devices are a building block of the wireless BCI system, extending their applicability from everyday assistance to PMFR (Minguillon et al., 2017). Moreover, with the gradual deployment of 5G wireless technology, the wireless EEG system can become more efficient in device-to-device communication with fewer artifacts, favoring the acceptability and usability of the wireless closed-loop BCI system developed in the future (Shakhakarmi, 2016).

#### 3.3.3. Electrode-tissue interface technologies

**Figure 4:**
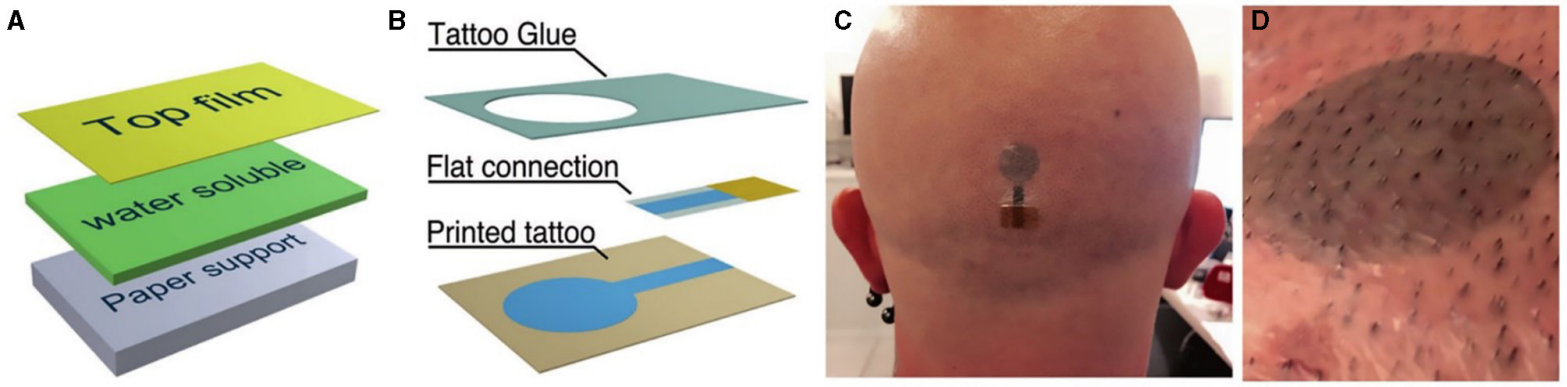
Temporary tattoo electrodes (TTEs) for EEG. **(A)** The layered structure of the temporary tattoo paper permits the release of the top film on which electrodes are fabricated. **(B)** Expanded view of an all-polymer printed TTE. **(C)** A TTE released on the scalp. **(D)** Close-in view of a TTE released on the scalp after 12 h from application.

Currently, the main obstacle to the clinical use of BCI systems is that the signal from the neuronal activity recorded by the electrodes can change over time (Perge et al., 2013). This is mainly due to small movements of the electrodes relative to the surrounding brain tissue, cell loss, and scar tissue effects. In addition, the volume conduction effect of the skull can also lead to inaccurate source localization. With respect to this concern, many advances have been made in electrode-tissue interface technology. Conventional EEG electrodes are wired wet electrodes that require the application of gel to ensure low impedance levels (<10 *kohm*). However, the conductive gel will dry out within a few hours, making the performance of the electrodes become unstable over time (Ferree et al., 2001). In addition, conventional electrodes are cumbersome and rigid, which is very uncomfortable for the patient during placement. To improve the above disadvantages, researchers have made many efforts. Current wireless EEG system usually uses dry electrodes, which needs shorter installation time and higher comfort (Hinrichs et al., 2020). Ultrathin-film devices that can laminate directly on the skin are a hot topic in dry electrode technology. The advantages of this technology include the ability to create a stable and accurate connection to the skin, as well as ease of design and production (Nawrocki et al., 2018; Tian et al., 2019). Temporary tattoo electrodes (TTEs) are one of these attempts. They are made of organic material that adheres well to the skin and does not cause significant discomfort to the patient (Ferrari et al., 2020). A newer, substrate-free, tattoo-like electrode system arranged the tattoo electrodes as transformable, filamentous serpentine lines that offered the benefits of softness and breathability for signal acquisition over a large area (Wang et al., 2020; Figure 4).

## 4. Triggering motor commands in BCI mediated PMFR

One of the main problems of BCI-mediated PMFR is how to conveniently start and operate the BCI system. The initiation of motor commands at the start node is critical for the operation of the closed-loop BCI. For PMFR, the BCI is designed to stimulate ipsilesional activity during movement of the affected limb. In stroke patients, however, the excitability of the cortex in the stroke area can also be further reduced by interhemispheric and intercortical inhibition (Xu et al., 2019). Therefore, a lack of sufficient motor commands usually degrades BCI performance significantly during PMFR. To solve the above problems, the functional design of BCI should be necessary to include the strategy of triggering self-paced motor commands, as well as the technology of auxiliary stimulation.

### 4.1. Motor imagery

As a self-paced mental practice to improve motor performance, motor imagery (MI) has gained prominence among post-stroke motor rehabilitation topics in recent decades, especially for robotic procedures. In stroke patients, the damaged motor network may prevent the BCI from decoding the motor signal in real time. Nevertheless, the MI process has a similar neural basis as the real motor performance (Cicinelli et al., 2006). Several studies have demonstrated the efficacy of MI coupled BCI technology in robotic rehabilitation. In the motor imagery brain-computer interface (MI-BCI) system, EEG signals detected during MI can be distinguished from those in the background at rest using specific algorithms and machine learning [e.g., Filter Bank Common Spatial Pattern (FBCSP)]. After the MI signal acquisition and calibration phase, these additional signals help the BCI to control the robot to assist the subject in moving the impaired limb toward the intended target (Ang et al., 2011). Clinical studies demonstrated that compared with standard BCI-driven robotic rehabilitation by coupling patients' motor intention and muscle control, the MI-BCI could improve motor recovery of the extremities after stroke. In addition, modulation of brain activity by transcranial direct current stimulation (tDCS) prior to MI-BCI shows tendencies to improve the efficacy of MI-BCI, suggesting that the MI-BCI could regulate cortical plasticity in an activity-dependent manner (Ang et al., 2015; Chew et al., 2020). One of the main factors limiting the use of MI in BCI is the identification of MI capability in stroke survivors to generate enough signals that EEG can detect and analyze (Zich et al., 2015). Many tools and methods and their combinations have been proposed to assess MI capability, including self-report questionnaires, mental chronometry, physiological indices, and EEG measurements (Madan and Singhal, 2014). However, due to the subjectivity of self-report and differences in individual characteristics, the reliability of these instruments is still under debate, which still poses challenges for appropriate participant selection and further use of MI for BCI control. Interestingly, some aspects of the subject's emotional status, such as confidence and attitude, may influence the results of MI assessment, suggesting that the MI may encompass more complex cognitive processes that need to be further explored (Marchesotti et al., 2016; MacIntyre et al., 2018).

In stroke survivors who have completely lost the motor functions of their limbs, the MI can still be remedied through neurofeedback introduced by the environment. The importance of this process is not only in providing motor signals to the BCI, but may also promote reorganization of the cortex by optimizing neuroplasticity after stroke. However, the *post-hoc* analysis is still rare. In a broader sense, MI includes multidimensional and multimodal constructs, such as visual-objective imagination, spatial imagination, kinesthetic imagination, etc. (Guillot and Collet, 2010). However, the concept of these neurally dissociable processes derived only from different studies on MI. It's not reasonable to divide MI into these processes separately (Kozhevnikov et al., 2005; Blajenkova et al., 2006).

### 4.2. BCI-combined brain stimulation

**Figure 5:**
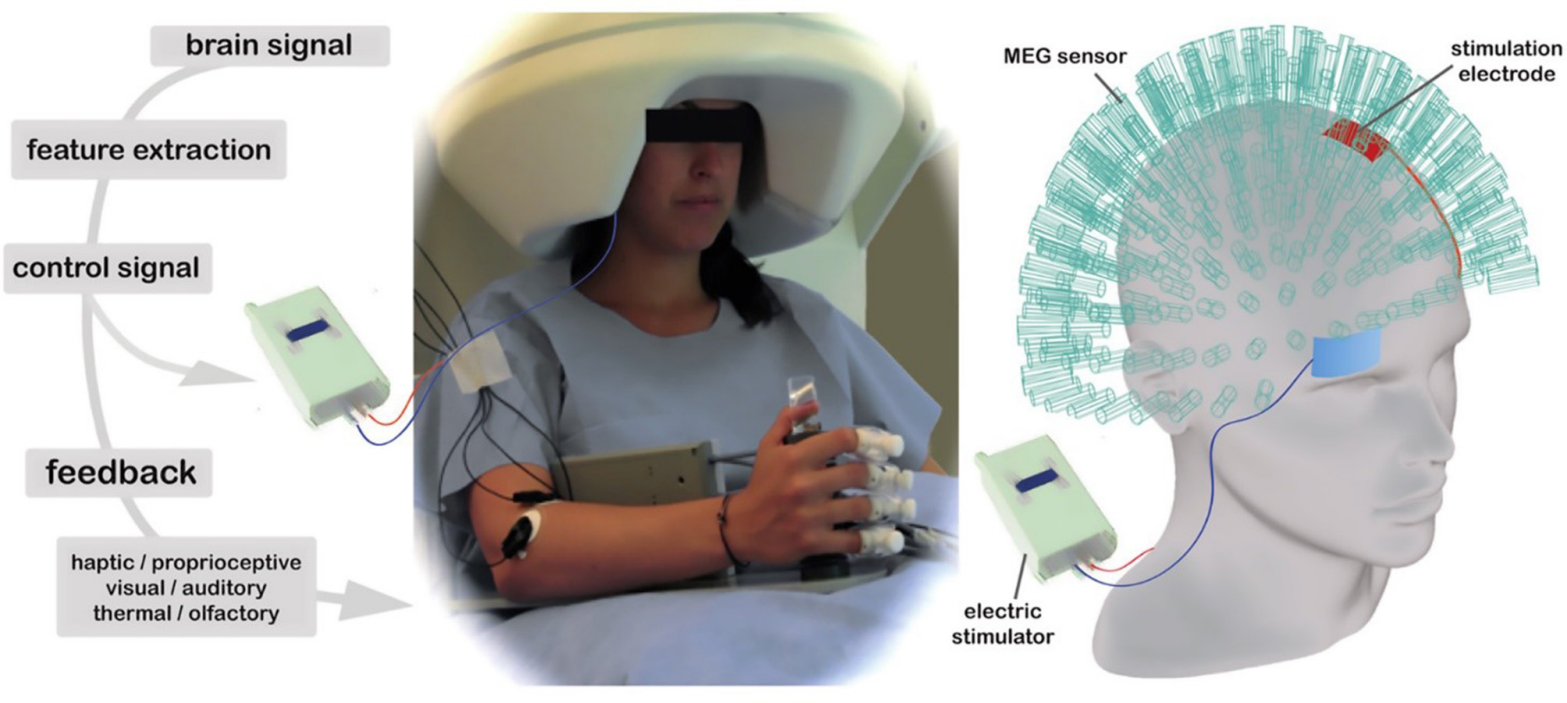
This design uses a 275-sensor whole-head MEG to record neuromagnetic brain activity during tDCS stimulation, with electrodes placed in the classic unilateral M1 montage. This set-up is used in conjunction with BCI visual feedback in the form of a computer game and sensorimotor feedback via a robotic hand orthosis that opened as target oscillations increased.

One of the prevailing neuroscientific models of PMFR is to provide excitation of the lesioned hemisphere with simultaneous inhibition of the non-lesioned hemisphere (Sung et al., 2013). Additional stimulations of the neuromotor control system at different nodes in the closed-loop of BCI-mediated PMFR have recently attracted interest. These stimulations can be both invasive and noninvasive, facilitating the accurate operation of BCIs as well as enhancing motor recovery after stroke through activity-dependent cortical plasticity (Liew et al., 2014). Compared with invasive methods, non-invasive brain stimulation (NIBS) such as transcranial direct current stimulation (tDCS) and repetitive transcranial magnetic stimulation (rTMS) is more feasible and suitable for BCI adaptations by modulating the brain cortical excitability with long-lasting effects (Figure 5; Table 1).

**Table 1:** Comparison of BCI types for triggering motor commands in BCI-mediated PMFR.

**BCI type**	**Key indicators**	**Advantages**	**Disadvantages**
Motor imagery (MI-BCI)	EEG signals, FBCSP, tDCS	Neural basis in MI, clinical efficacy	MI capability assessment challenges
BCI-combined brain stimulation	rTMS, tDCS, optogenetics	Feasible, modulates cortical excitability	Optogenetics in preclinical stage
Strengthening external devices	Exoskeletons, neuro-prostheses	Promotes motor learning, user-friendly	Maturity of exoskeleton designs needed
Multimodal feedback	Tactile, proprioception, auditory, VR	Enhances motor learning, engagement	Need for tailored feedback strategies

#### 4.2.1. rTMS

The rTMS is one of the NIBS methods in which a magnetic field produces a continuous train or periodic trains of pulses to increased (high frequency, >3 Hz) or decreased (low frequency, <1 Hz) the cortical excitability. Although there is a potential risk of epilepsy with the use of rTMS in the acute phase of stroke, more positive effects of rTMS on motor recovery have been observed in recent studies (Kubis, 2016). It is typically recommended to use low frequency rTMS intervention in stroke patients with an unstable medical condition during acute stage, which aims to inhibit the excitability of unaffected cerebral hemisphere. Once the damaged hemisphere was relatively stabilized, reverse treatment procedure was delivered to the patients in order to activate the lesion side by virtue of the high frequency stimulation. As an add-on intervention, rTMS aims to up-regulate ipsilesional excitability through facilitatory stimulation on the ipsilesional hemisphere or inhibitory stimulation on the contralesional hemisphere, which can be combined with BCI to promote motor recovery (Johnson et al., 2018).

#### 4.2.2. tDCS

In tDCS, weak direct electrical currents are used to alter the firing threshold of the neuronal membrane in specific parts of the brain, modifying spontaneous activity. Depending on the direction of the current (anodal or cathodal), it can decrease or increase cortical excitability (Nitsche and Paulus, 2000; Thair et al., 2017). Compared to rTMS, the tDCS device is much smaller and portable. The tDCS can be used as a priming tool for closed-loop BCI to facilitate the MI process to strengthen motor command (Ang et al., 2015). A recent study showed that tDCS prior to BCI-based motor imagery training reduced resting motor threshold (RMT) in the ipsilesional M1 cortex and short intracortical inhibition (SICI) in the contralesional M1, helping to improve motor function in chronic stroke patients (Chew et al., 2020). Another promising development is that, apart from conventional tDCS with fixed intensity and duration, tDCS can be triggered and modulated by online EEG, forming a closed-loop tDCS-based system. The EEG-tDCS closed-loop system could promote motor learning with real-time regulated tDCS (Leite et al., 2017).

#### 4.2.3. Optogenetics

One of the drawbacks of currently clinically available brain stimulation techniques is that they activate mixed populations of neurons and astrocytes within less focal brain areas (Zhao and Rempe, 2010). Compared with rTMS and tDCS, optogenetics can induce selective excitation or inhibition in specific subtype neurons which helps to bypass neural circuits impaired by stroke (Böhm et al., 2020). More recently, optogenetic stimulation has come into the spot-light in the field of post stroke recovery. A study in nonhuman primates shows that noninvasive optogenetic stimulation can activate selective neurons in the primary motor cortex to generate forelimb movements and, in particular, induces long-lasting modulation for voluntary movements (Ebina et al., 2019). However, even though optogenetic stimulation is still at the preclinical exploration stage, the noninvasive improvements of this young technology pave the way for its application in more precise control of BCI (Hira et al., 2009).

## 5. Strengthening of external devices in BCI-mediated PMFR

The robotic devices, such as exoskeletons and manipulators (end-effector devices), have been developed as independent and passive machines to perform therapies that require high intensity repetition. However, as the effector node in closed-loop BCI, these external devices can be controlled by stroke patients through the BCIs, according to the assistive and rehabilitative needs of the users (Alia et al., 2017). Compared with assistive use, the rehabilitative use of these devices is more meaningful for post stroke patients because they promote the remodeling of the sensorimotor system through the Hebbian-like plasticity mechanisms (Ward, 2017; Biasiucci et al., 2018). In recent years, new functional designs and device-controlling technologies in BCI systems have been increasingly introduced into the clinical practice of PMFR, which has significantly increased the utility of these external devices.

### 5.1. Promoting the user-friendliness

Compared to the stiff interaction mode of early immature robotic devices for PMFR, the design of rehabilitation robots has recently become more user-friendly. In terms of the functions they perform, limb rehabilitation assistive robots can be divided into two categories: (i) exoskeletons for the restoration of the functions of limbs and (ii) neuro-prostheses for replacement of the disabled limbs. However, for PMFR, a device to restore limb motor function by helping the motor cortex to acquire motor control function through motor learning is more important than a replacement of limbs. It has been shown that repetition and prolonged training can be beneficial for the recovery of limb strength, but have little effect on the reorganization of the cortical map (Remple et al., 2001; Swain et al., 2003). Therefore, unlike prostheses, the design of exoskeletons for motor recovery requires the analysis and consideration of critical issues, such as embodiment with the paretic limbs and augmentation of the adaptive reorganization of motor cortex after stroke. With the continuous advances in materials science and design concepts, the user-friendliness of peripheral robots for post-stroke rehabilitation is improving, such as the soft robotic gloves, elbow sleeves, ankle exoskeletons, and a whole limb exosuit (Laschi et al., 2016; Walsh, 2018). Several feasibility studies of BCI-based wearable devices have been conducted, although the results are still somewhat heterogeneous, they offer scope for exploring future applications (Koh et al., 2017; Cheng et al., 2020). Many sophisticated exoskeleton designs have been performed to facilitate BCI-assisted therapy, optimizing device features such as higher comfort, ease of use, safety, and energy savings during rehabilitation (Awad et al., 2017).

### 5.2. Reinforcing the control

Ideally, in a closed-loop BCI system, the control strategy of external devices should be in line with the theoretical basis of voluntary motor control in the CNS. In addition to the development of the design of the external devices, great interest has been focused on the control strategies such as integrating feedback sensors and electrical stimulators into robotic devices. In several robotic assistive devices, feedback elements have been incorporated into the wearable exoskeletons, providing active feedback (tactile, vibrotactile, or force) to the limbs of the user. However, which feedback would be most important and how this feedback should be provided is still under investigation. Nevertheless, in stroke patients with impaired proprioception and tactile sensation, the missing sensory information can be compensated by the feedback, which is important both for the completion of a specific task and for the recruitment of the motor control circuit (Ben-Tzvi and Ma, 2014; Ma et al., 2015). In BCI systems, the exoskeletons can also be integrated with transcutaneous functional electrical stimulation (FES). Compared to conventional FES, BCI-guided FES can induce appropriately timed neuromuscular stimulation through BCI command that reduces spasticity, improves range of motion and muscular synergy, and induces durable motor recovery by promoting targeted neuroplasticity through sensory feedback (Mazzoleni et al., 2017; Moon et al., 2017; Biasiucci et al., 2018). Although the design of BCI-driven assistive devices is still not yet mature, it is developing very fast, many promising strides have been made, such as the wireless control, the gaze-based control, and the out-of-body control, etc. (Penaloza and Nishio, 2018; Kim et al., 2019). In the future, external devices as closed-loop effectors may need to be combined into a multifunctional platform that could provide real-time motion analysis, epidural electrical stimulation, sensory feedback, and personalized adaptive support, with the goal of not only strengthening control but also promoting motor learning through neuroplasticity.

## 6. Closing the loop with multimodal feedback in BCI mediated PMFR

The neurofeedback is a key point that links the two ends of an open-loop to a closed one during the operation of a closed-loop BCI system. In the process of BCI-mediated PMFR, the effect of neurofeedback can be two-fold: (i) close the sensorimotor loop with self-regulation, thus facilitating the control of external assistive robotics (ii) activate the plasticity system, thus promoting the reorganization of the motor cortex. The neurofeedback can induce endogenous neural stimulation to facilitated motor output. For example, the same object can evoke different voluntary actions through its physical properties and the behavioral salience observed by the observer. The somatic sensory input from body receptors can be a teaching signal during motor learning, presenting as feedback-error learning and supervised learning. Learning a motor skill can continually reorganize or shape a stroke survivor's motor map. Animal and human experiments have demonstrated that neural activity can be self-regulated through neurofeedback. A prevailing closed-loop modality for BCI-mediated PMFR is often based on non-invasive EEG, it involves three steps: (i) EEG collects neural activation signals in the M1 region to control the surrounding prostheses or exoskeletons, (ii) terminal devices performance generate behaviors as the source of feedback to change the firing rates of a population of cortical neurons, (iii) learned control of multiple neurons is presented simultaneously to regulate EEG amplitudes, which enhances internal processing to facilitate control of the external device or paretic limb motor activity during task-specific actions (Collinger et al., 2013; Bouton et al., 2016; Figure 2). Moreover, neurofeedback-based close loop training can also be used to regulate emotion processing, such as strengthening connectivity between cognitive control areas, and lead to behavioral improvements (Koush et al., 2017). The main advantage of the EEG-based BCIs with an established closed-loop is that they can support the completion of limb motor function while playing a role in strengthening motor control by improving brain plasticity. Regarding the hallmarks of closed-loop BCIs, they may be consistent with the same neural mechanisms that operate in voluntary movement control, which is consistent with the “fire together, wire together” principle in Hebbian learning (Soekadar et al., 2015).

### 6.1. Different types of feedback can be used to close the BCI loop

One of the main goals of neurofeedback is to train users to adapt to the BCI task by providing specific cues to task-related brain activity. In addition to the content of the feedback, the way in which the feedback is presented also has a major impact on its effect (Pillette, 2019). The following feedback modalities have been explored (Table 2).

**Table 2:** Comparison of feedback modalities in BCI-mediated PMFR.

**Feedback modality**	**Characteristics**	**Advantages**	**Disadvantages**
Tactile sense	Thermal, pressure, vibration	Enhances sensory perception	Limited feedback options
Proprioception	Limb position and force feedback	Improves motor control, kinesthetic	Requires specialized devices
Auditory	Speech, tone, music	Motivational, supports motor imagery	Over-familiarization may reduce motivation
Visual display	Abstract signals, simulated hand, VR	Enhances engagement, motion visualization	Variable effectiveness across users

#### 6.1.1. Tactile sense

In the closed-loop system, the tactile sense can help the subject to perceive hardness, texture, temperature, and vibrational stimuli from the environment. Within the tactile interfaces between tactile receptors of the skin and the external tactile-providing device, the tactile feedback can be integrated into the closed-loop of BCI mediated PMFR (Chatterjee et al., 2007; Cincotti et al., 2007). The most commonly used tactile provider are lightweight and wearable devices that can generate feedback from thermal cues, contact pressure, mechanical vibration, and electrotactile (Jones and Sarter, 2008; Gabardi et al., 2016).

#### 6.1.2. Proprioception

The function of proprioception or kinesthetic feedback for users in the BCI-mediated loop is mainly to know the position of the body in space and the force on the limbs (Williams, 2015; Pacchierotti et al., 2017). The sensation can be generated from force-feedback devices embodied in external devices (e.g., grounded devices, exoskeletons), as well as muscle contraction actuated by FES (Pfeiffer and Rohs, 2017).

#### 6.1.3. Auditory

Auditory feedback has been shown to support PMFR and activate plasticity. The auditory feedback can be presented as speech, pure tone, and music with different sound speaker arrangements to influence motor imagery performance (McCreadie et al., 2014). The auditory feedback can also use the features of music (e.g., the volume, the tempo) to help users to operate the BCI in a closed-loop (Kellaris et al., 1996; Daly et al., 2014). Of note, the decreased motivation induced by over-familiarization of the music should be considered when using music properties as neurofeedback (Nijboer et al., 2008). In turn, however, various music-induced emotions could support patient engagement in the BCI system.

#### 6.1.4. Visual display

##### 6.1.4.1. Abstract signal

More recently, neurofeedback has been introduced to remedy motor imagery-based BCI training in PMFR. The expression of the feedback is usually an abstract signal (a moving bar or a ball on the screen) that provides the patient himself the information on how good his performance of the MI tasks (Zich et al., 2017).

##### 6.1.4.2. Simulated hand

In upper limb PMFR, the simulated hand has been used more frequently compared to the abstract signal as an embodied neurofeedback that resembles the content of the MI act. The simulated hand can be a rubber hand, its VR-based derivatives, and a movable robotic hand that more closely resembles the shape and function of a human hand (Braun et al., 2014; Kalckert and Ehrsson, 2014; Pichiorri et al., 2015; Spychala et al., 2020). However, many state-of-the-art prosthetic hands have not yet been used in this field, which provides more room for future research (Laffranchi et al., 2020).

#### 6.1.5. Virtual reality

Virtual reality (VR) is an immersive computer-based technology that places the user in simulated environments with real objects and events. There is growing evidence that VR may promote PMFR in combination with both conventional therapy and BCI-mediated therapy (Silvoni et al., 2011; Fluet and Deutsch, 2013). As a node to generate the feedback of the closed-loop system of BCI, VR made the patient immersed in different scenes with the feeling of embodiment of the virtual environment. The VR system often provides multimodal feedback to the subject, such as visual, auditory, tactile, and so on. The most commonly used VR strategy is motion visualization, which represents the patient's behavior and provides performance feedback in a virtual environment with certain contextual information. In this circumstance, the motion can be represented by a virtual body or a non-anthropomorphic graphic in a 2D or 3D environment (i Badia et al., 2012; Ferreira dos Santos et al., 2016).

### 6.2. Neurofeedback-induced effects in closed-loop BCI system

Recently, the closed-loop BCI system using neurofeedback through online pattern has been shown to regulate the learned-control effect in PMFR (Cano-De-La-Cuerda et al., 2015). The self-regulation of neural activity through neurofeedback training has been found in rodents, nonhuman primates and humans (Schafer and Moore, 2011; Collinger et al., 2013; Clancy et al., 2014). The consequence of this self-regulation can be represented as changes in intracortical neuronal synchronization that facilitate the output of EEG-based BCI (Hanslmayr et al., 2005; Blefari et al., 2015). Moreover, neurofeedback training can also exert long-term changes in the intrinsic functional connectivity in the visuo-spatial-motor network, even more than 2 months after the training (Megumi et al., 2015). These explorations demonstrated that the meaning of this kind of brain functional changes is not only to help patients to operate the BCI to control external devices within the closed-loop system, but rather to activate the neuroplasticity along with a motor learning process (Sitaram et al., 2017).

Another important effect of neurofeedback is the control of negative emotions in the subject in the closed-loop system of BCI-mediated PMFR. It is supposed that subjects' motivation and engagement may be more sustained during robotic training than in conventional therapy (Jeunet et al., 2015). Efforts often center on concerns of the design of external robotic devices, but another important factor in ensuring the implementation of robotic-mediated rehabilitation is the neuropsychological response of the human side. There is growing evidence that people's interactions with multimodal feedback or environment (e.g., VR) are more enjoyable and motivating than interaction with robots alone (Mladenović et al., 2017; Baur et al., 2018). The motivational state depends on the circumstance in which the subject was, for example, a post-stroke patient may reach to grab a cup simply because of thirst, otherwise the patient may just fulfill the entire set of actions to achieve a goal set by the therapist. Consequently, the purpose may affect the firing pattern of neurons in the inferior parietal lobe, which has been demonstrated in monkey tests (Fogassi et al., 2005). In addition, the patient's engagement in the stroke rehabilitation process often depends on behavioral factors as the patient's motivation in task-directed training, trust in the effectiveness of therapist or equipment, and understanding of mechanisms of rehabilitation protocols, etc. These cognitive aspects of motor impairment after stroke often coexist in stroke survivors and, notably have a potential impact on the outcome of neurological rehabilitation. Thus, a pre-procedural session to assist the users to comprehend and engage in the BCI system, protocol and mechanism is mandatory (Remsik et al., 2018).

## 7. Outlook

While closed-loop technology based on BCI has gradually matured in the application of PMFR, future research endeavors are still needed to prioritize the following areas: (1) Understanding Compensatory Motor Control Mechanisms in Post-Stroke Patients: Existing research has provided insights into compensatory motor control mechanisms in post-stroke patients, such as neural reorganization and adaptive processes. However, future research should delve deeper, utilizing tools like neuroimaging, biosensors, and computational models to precisely identify and quantify these mechanisms. Integration of these findings into the BCI system will be crucial for achieving more effective motor rehabilitation. (2) Customizing Multimodal Feedback for Individual Patients: In current research, efforts have been made to explore the customization of multimodal feedback based on individual patient conditions and needs. These customization methods can adapt feedback based on the emotional state, sensory abnormalities, and cognitive function of patients. Leveraging machine learning and patient-specific data, such as neural signatures and behavioral responses, will enable a more personalized approach to BCI-mediated PMFR. (3) Advancements in BCI-Compatible Brain Stimulation Techniques: Significant progress has been made in developing BCI-compatible brain stimulation techniques. These techniques leverage neurofeedback and real-time monitoring to optimize stimulation timing and intensity. Additionally, advancements in non-invasive brain stimulation modalities, such as transcranial magnetic stimulation (TMS) and transcranial direct current stimulation (tDCS), are becoming increasingly integrated into BCI systems for neuroplasticity induction. (4) Enhancing Brain Motor Signal Extraction Techniques and Devices: Recent research has focused on improving brain motor signal extraction techniques and devices. Advanced signal processing algorithms, including deep learning approaches, have reduced noise and enhanced signal reliability. Portable and wireless EEG (electroencephalogram) devices are now more accessible, allowing for real-world applications of BCI-mediated PMFR with greater convenience and improved signal quality. (5) Developing Customized Soft, Wearable Exosuits: Research has led to the development of soft, wearable exosuits tailored to individual functional needs. These exosuits incorporate flexible materials and ergonomic designs to ensure comfort and ease of use. Integration with BCI technology involves optimizing the communication interface between the exosuit and the BCI system, allowing for seamless control of assistive devices tailored to each patient's motor rehabilitation requirements.

## Author contributions

JM: Writing—original draft. ZX: Writing—original draft, Writing—review and editing. YQ: Writing—review and editing. TZ: Methodology, Writing—original draft, Writing—review and editing.
